# Gene expression profiling of immunomagnetically separated cells directly from stabilized whole blood for multicenter clinical trials

**DOI:** 10.1186/s40169-014-0036-z

**Published:** 2014-11-13

**Authors:** Martin Letzkus, Evert Luesink, Sandrine Starck-Schwertz, Marc Bigaud, Fareed Mirza, Nicole Hartmann, Bernhard Gerstmayer, Uwe Janssen, Andreas Scherer, Martin M Schumacher, Aurelie Verles, Alessandra Vitaliti, Nanguneri Nirmala, Keith J Johnson, Frank Staedtler

**Affiliations:** 1 Biomarker Development, Novartis Institutes for BioMedical Research (NIBR), Basel, Switzerland; 2 Biomarker Development, Novartis Institutes for BioMedical Research (NIBR), Cambridge, MA, USA; 3 Scientific Capability Development, Pharma-Development, Novartis Pharma AG, Basel, Switzerland; 4 Miltenyi Biotec GmbH, Bergisch Gladbach, Germany; 5 Spheromics, Kontiolahti, Finland

**Keywords:** Cell sorting, Transcriptomics, Clinical

## Abstract

**Background:**

Clinically useful biomarkers for patient stratification and monitoring of disease progression and drug response are in big demand in drug development and for addressing potential safety concerns. Many diseases influence the frequency and phenotype of cells found in the peripheral blood and the transcriptome of blood cells. Changes in cell type composition influence whole blood gene expression analysis results and thus the discovery of true transcript level changes remains a challenge. We propose a robust and reproducible procedure, which includes whole transcriptome gene expression profiling of major subsets of immune cell cells directly sorted from whole blood.

**Methods:**

Target cells were enriched using magnetic microbeads and an autoMACS® Pro Separator (Miltenyi Biotec). Flow cytometric analysis for purity was performed before and after magnetic cell sorting. Total RNA was hybridized on HGU133 Plus 2.0 expression microarrays (Affymetrix, USA). CEL files signal intensity values were condensed using RMA and a custom CDF file (EntrezGene-based).

**Results:**

Positive selection by use of MACS® Technology coupled to transcriptomics was assessed for eight different peripheral blood cell types, CD14+ monocytes, CD3+, CD4+, or CD8+ T cells, CD15+ granulocytes, CD19+ B cells, CD56+ NK cells, and CD45+ pan leukocytes. RNA quality from enriched cells was above a RIN of eight. GeneChip analysis confirmed cell type specific transcriptome profiles. Storing whole blood collected in an EDTA Vacutainer® tube at 4°C followed by MACS does not activate sorted cells. Gene expression analysis supports cell enrichment measurements by MACS.

**Conclusions:**

The proposed workflow generates reproducible cell-type specific transcriptome data which can be translated to clinical settings and used to identify clinically relevant gene expression biomarkers from whole blood samples. This procedure enables the integration of transcriptomics of relevant immune cell subsets sorted directly from whole blood in clinical trial protocols.

## Background

There is an ever-increasing demand for the discovery, validation, and application of clinically useful molecular biomarkers that enable patient stratification, diagnosis, monitoring of disease progression, or a better understanding of drug response (safety and efficacy) [1]-[3]. Gene expression biomarkers have been investigated in various diseases such as autoimmune diseases [4],[5], cancer [6]-[8], neurological diseases [9],[10], infections [11],[12] and in transplantation [13],[14]. Whole blood or peripheral mononuclear cells (PBMCs) have long been the preferred surrogate tissues for clinical transcriptomics in biomarker research for two major reasons: Firstly, blood is an easily accessible human tissue, and secondly, robust sampling methods of whole blood (e.g. PAXgene Blood RNA Tubes) [11],[12],[15]-[17] and PBMCs [18],[19] are well established. As is the case with any tissue sample with mixed cell population, whole blood or PBMC transcriptome analysis approaches are sensitive to not only to variation in cell-type composition of the sample, but also to the physiological state of the donor, and technical factors such as sample storage [20],[21]. It is therefore important to understand these factors and consider them prior to the study.

Gene expression analysis of isolated cell populations instead of whole blood or PBMC circumvents the need to adjust for potential cell composition variation. Cell populations need to be sorted from the mixture of blood cells by means of a complex cell sorting technology, which would need to be translated to clinical trial settings. The decoupling process needs to guarantee the quality of the source of the cells from limited access to sophisticated cell sorting equipment. Variation in the duration of transport to central laboratories needs to be considered by ensuring sample stability and proper storage conditions. Only a high level of reproducibility assures comparability and reliability of analysis results within a set of samples from a patient and within patient groups.

We assessed a cell separation technology using antibody-coated paramagnetic beads for sorting cells directly from human whole blood under conditions representing a clinical trial. Positive cell selection protocols based on Miltenyi Biotec¿s whole blood microbeads were considered for the following reasons: 1) Miltenyi Biotec¿s autoMACS Pro Separator is capable of sorting approximately 10^6^ cells per second in a rapid, semi-automated fashion [22]. Rapid throughput minimizes the potential perturbation of the transcriptome. The time required for preparative sorting for transcriptomics analyses is sample dependent. 2) As Lyons et al. reported, the cell purity after positive selection is higher than that after negative selection [23]. 3) Magnetic cell sorting can be performed in a multiplex format, thus further decreasing sorting time when a number of cell types are being purified.

**Figure 1:**
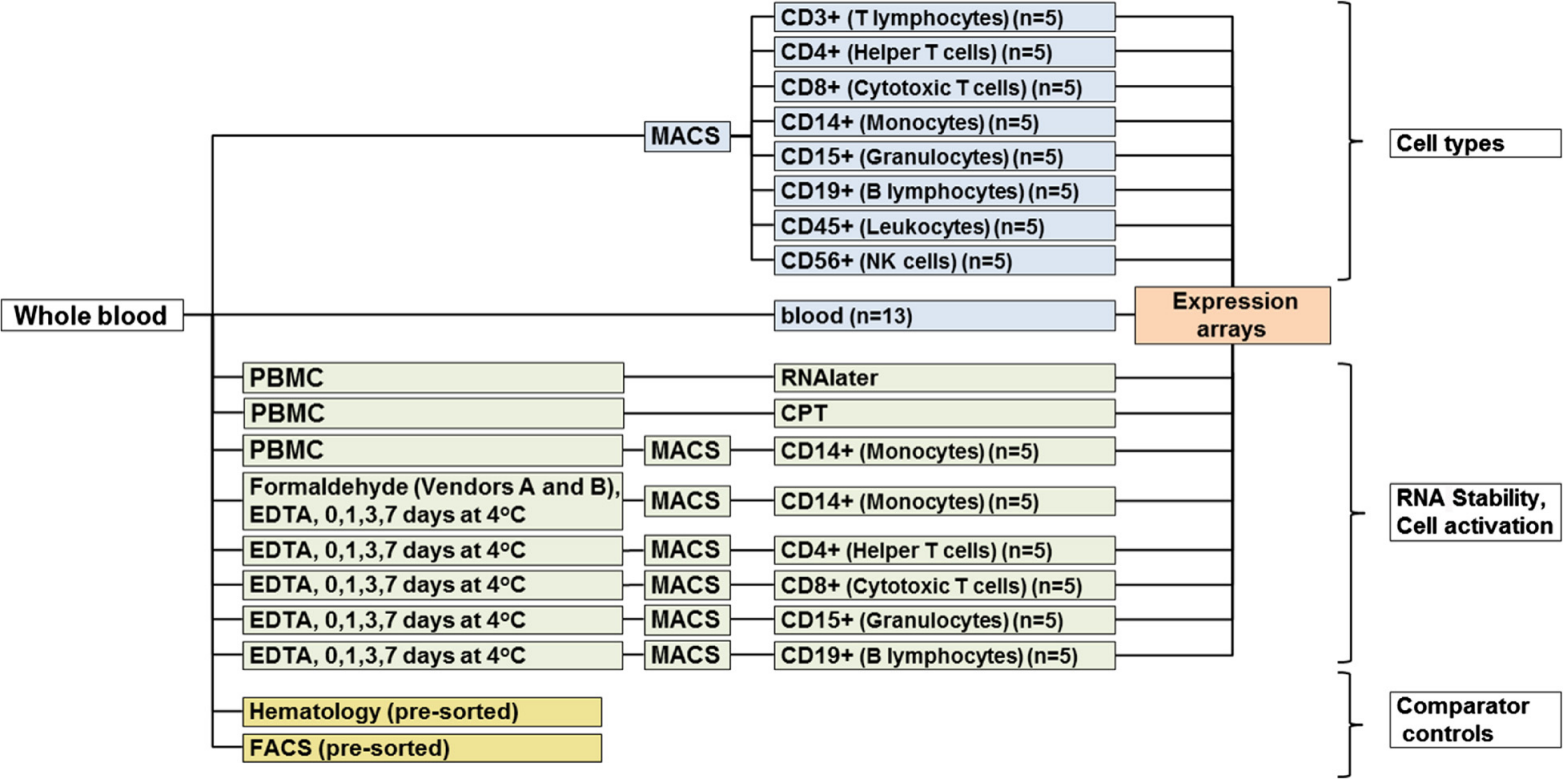
**Experimental study design.** The study design consisted of three major components: (1), in blue: gene expression analysis of a panel of eight different cell types positive selected by MACS directly from whole blood followed by GeneChip expression arrays. (2), in green: RNA stability tests and RNA preservation methods, and (3), in yellow: hematology and pre- and post-sorting FACS analysis as controls

Three major components were included in the experimental study design (Figure 1). One component addressed whole transcriptome analysis of a panel of eight different cell populations isolated directly from 6 mL whole blood, each, collected into EDTA containing tubes using a positive selection with the autoMACS Pro Separator, and of PBMCs prepared from BD CPT (Becton Dickinson Cell Preparation Tubes). EDTA, ethylendiaminetetraacetic acid, is a chelating molecule, capable of binding bivalent metal ions such as Ca2+, Fe2+ and Mg2+. As Mg2+ ions are important cofactors for RNA-degrading RNAses, the addition of EDTA provides a greater RNA stability. The purified cells, representing eight different abundant blood cell populations (CD14+ monocytes, CD3+, CD4+, or CD8+ T cells, CD15+ granulocytes, CD19+ B cells, CD56+ natural killer (NK) cells, CD45+ pan leukocytes) are well suited for further transcriptome analysis. Another variable considered in this study included the evaluation of different RNA stabilization and preservation methods in order to uncouple the site of blood draw from the actual cell sorting process. A volume of 6 mL was chosen, as this is a volume that can realistically be obtained in clinical trials without exceeding the possible total amount of blood that can be drawn in the context of other measurements. In yet another component, we performed clinical hematology counts and pre- and post-sorting flow cytometric analysis of the sorted cell populations to measure the clinical relevance and efficiency of the experimental sampling conditions.

Here we present the results of a feasibility study and discuss the possibilities and limitations of gene expression analysis of sorted cells in future clinical studies.

## Methods

### Study subjects and blood samples

Blood samples were obtained prospectively from 45 adult, consented Caucasians (18 females and 27 males) from a donor pool of healthy volunteers. Venous blood samples were drawn from the cubital region using evacuated tubes containing K_2_-EDTA as anticoagulant (BD¿ Vacutainer, Becton Dickinson, Franklin Lakes, New Jersey, USA) or as control samples into PAXgene® Blood RNA Tubes (Qiagen, Hilden, Germany).

Samples from a subset of 23 donors (10 females and 13 males) were used for hematology and flow cytometry evaluation. Those were the only samples used for cell sorting.

### Hematology

Blood samples were stored at 4°C for up to 6 hours after venipuncture until analysis on a Siemens ADVIA 120 analyzer (Siemens Healthcare Diagnostics, Erlangen, Germany). The analyzer was calibrated and controlled according to the manufacturer¿s user manual. Only original reagents produced by the manufacturer were used and maintenance had been performed at the recommended intervals.

### Magnetic MicroBead-based cell sorting

Magnetic cell separation by MACS® Technology is based on small superparamagnetic microbeads that are bound to a highly specific antibody against a particular cell marker, thus allowing for magnetic labeling of individual cell types. Separation occurs in a MACS Column which induces a high-gradient magnetic field (~0.6 Tesla) when placed in a MACS Separator.

Cell separation directly from whole blood was performed using the respective Whole Blood MicroBeads (Miltenyi Biotec) as recommended by the manufacturer. A more detailed description of the experimental procedure can be found in the Additional file 1: Supplementary material.

### Flow cytometry

Whole blood samples and corresponding sorted cells were stored at 4°C until analysis on a MACSQuant® Flow Cytometer (Miltenyi Biotec GmbH).

Anti-human monoclonal antibodies against CD3+ T-lymphocytes, CD4+ helper T-cells, CD8+ cytotoxic T-cells, CD14+ monocytes, CD15+ granulocytes, CD19+ B-lymphocytes, CD45+ leukocytes and CD56+ NK cells were obtained from Miltenyi Biotec GmbH. For flow cytometry 250 ?L suspensions of isolated cells were incubated with 10 ?L of corresponding antibodies for 10 minutes at 2-8°C in the dark. The cells were washed and re-suspended in 500 ?L analysis buffer and afterwards immediately analyzed on a MACSQuant Flow Cytometer using MACSQuantify¿ Software. Data were further processed with FlowJo® software (www.flowjo.com).

### Total RNA extraction

The total RNA from the sorted and lysed cells was isolated with the RNeasy Mini Kit (Qiagen, Hilden, Germany) and from whole blood with the PAXgene Blood RNA Kit (Qiagen, Hilden, Germany) according to the manufacturer¿s recommendations. A more detailed description can be found in the in the Additional file 1: Supplementary material.

### Whole transcriptome analysis

Whole transcriptome analysis was conducted essentially as described by Lockhart et al. [24] on expression microarrays. The human genome U133 plus 2.0 array (Affymetrix, Inc.) was used. GeneChip arrays were scanned using a GS 3000 scanner (Affymetrix, Inc.).

Experiments were conducted as recommended by the manufacturer (GeneChip® Expression Analysis Technical Manual) using the cRNA labeling, GeneChip® hybridization, wash, and stain protocols (Affymetrix, Inc., Santa Clara, CA, U.S.A.). Quality control was performed by visual inspection and expert judgment. The primary raw data images, the .dat-files, were processed to .cel-files and to numerical signal values.

### Data analysis/Bioinformatics analysis

All gene expression analyses were performed in Partek® Genomics Suite¿, version 6.6 (www.Partek.com). Affymetrix .cel files were RMA background corrected, subjected to quantile normalization and scaled to a mean of 150. A custom Entrez gene-based CDF file was used for probe set condensation and annotation (http://brainarray.mbni.med.umich.edu/Brainarray/Database/CustomCDF/genomic_curated_CDF.asp). Cell type-specific gene signatures included those genes with a signal intensity of 200 or more in at least all but one sample of one cell type, but less than 100 in at least all but one of the samples of the other cell types excluding stabilized whole blood. The gene expression databases BioGPS (http://www.BioGPS.org) and Genevestigator (http://www.Genevestigator.com) were consulted for confirmation of cell type specific gene expression data. Gene groups for the perturbation score analysis were downloaded from www.SABiosciences.com. Unigene identifiers were mapped to Entrez identifiers, resulting in 502 probes of the custom, Entrezgene-based CDF file (Additional file 2: Supplementary table). Graphical presentation of data was performed using Prism GraphPad version 6.03 (www.graphpad.com).

## Results

### Hematology

Blood samples of a subset of subjects (10 females, 13 males) were analyzed on a Siemens ADVIA 120 analyzer (Siemens Healthcare Diagnostics, Erlangen, Germany). Time lag between venipuncture and analysis is considered not to affect cell counts [25],[26]. All cell counts were within the expected range for female and male healthy volunteers without overt pathological conditions affecting blood cell composition (www.Siemens.com/diagnostics) (Additional file 1: Table S2).

### Cell sorting experiments - total RNA yield and quality

Total RNA yield and quality were assessed for stabilized whole blood samples and for isolated cell populations. Cell separation from K_2_-EDTA anticoagulated whole blood samples started within 30 minutes after venipuncture.

Overall, the total RNA yields and quality were sufficient for all different cell types tested (Additional file 1: Figure S1). The highest mean RIN value was 9.65?±?0.18 for CD14+ cells, the lowest was 8.62?±?0.65 for stabilized whole blood. The lowest RIN value was 6.6 for one of 58 whole blood samples. All total RNA samples were processed further in Affymetrix DNA microarray experiments. The mean quantity of extracted RNA per 6 mL ranged from 0.35?±?0.12 ?g for CD56+ cells to 9.8?±?5.12 ?g for whole blood. The minimum amount of extracted total RNA was 0.113 ?g, the maximum was 24.6 ?g for a CD15+ samples and a blood sample, respectively.

### Cell sorting experiments - total RNA stability

**Figure 2:**
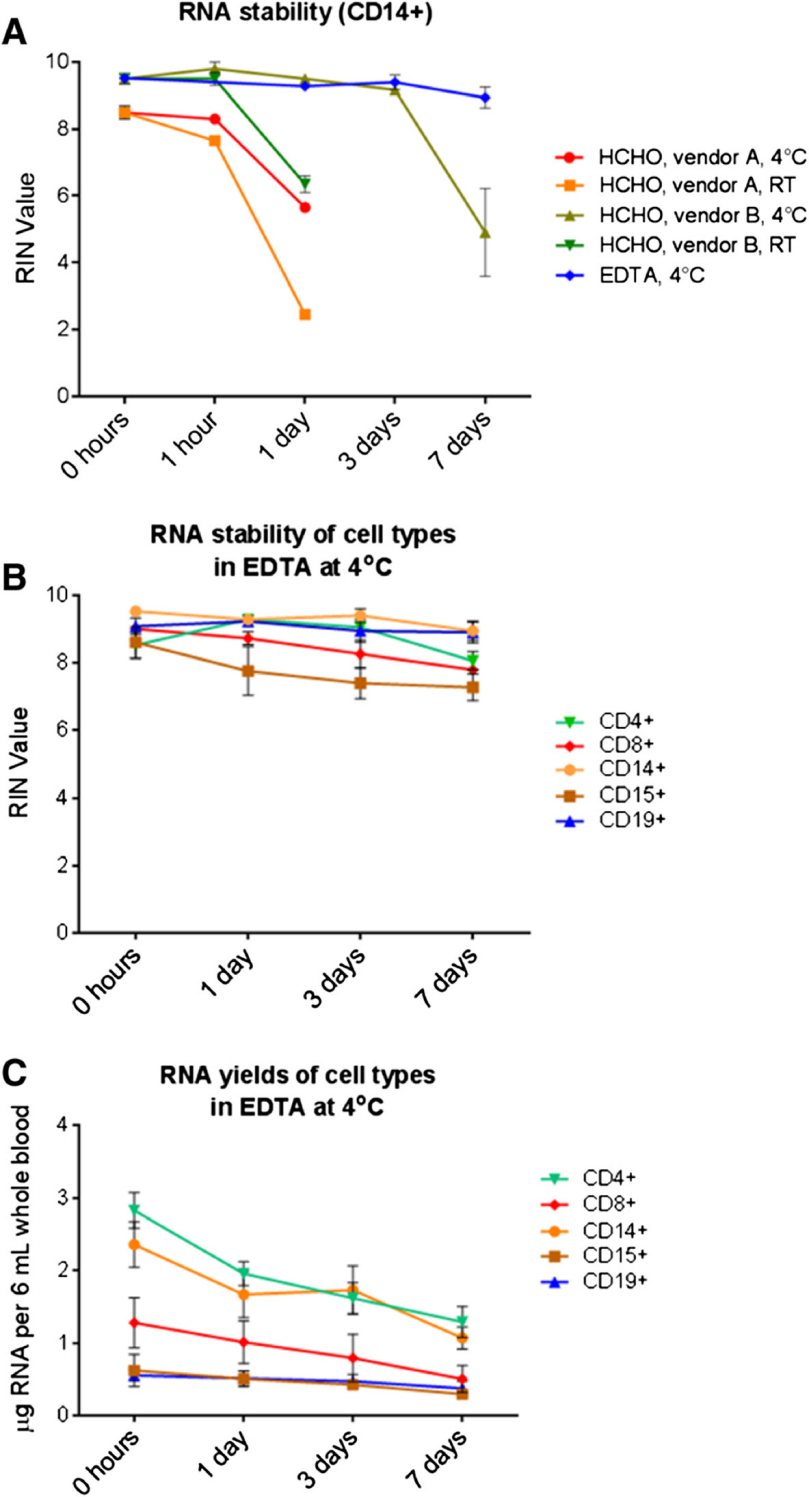
**RNA integrity and yield over time. (A)** The influence of formaldehyde (HCHO)-based and EDTA-based additives on RNA integrity, when whole blood was stored at ambient temperature or at 4°C for up to 7 days prior to sorting. EDTA-admixtures resulted in constantly high RIN values for the cell preparations up to 7 days of storage at 4°C. **(B)** All five tested cell types (N?=?5 each) showed a good total RNA quality when stored in the EDTA blood collection tubes at 4°C for up to 7 days prior to sorting. **(C)** Total RNA extraction for all five tested cell types (N?=?5 each) resulted in total RNA amounts sufficient for further downstream experiments even when stored in the EDTA blood collection tubes at 4°C for up to 7 days. In all panels, mean values per group and the standard error of mean are shown.

To address the influence of potentially stabilizing additives in blood collection tubes on the stability of the extracted RNA over time, an EDTA-based additive was compared to formaldehyde-based additives from two vendors, A and B. The collection tube with formaldehyde-based additive from vendor A is designed for stabilizing white blood cells for FACS analysis [27]-[31], the tube-type from vendor B is designed for stabilizing circulating tumor cells [25],[26],[32]. Whole blood was drawn and kept for up to 7 days at 4°C in the case of the EDTA-based tubes, and at either ambient temperature or at 4°C in the case of the formaldehyde-based tubes. CD14+ cells were isolated and total RNA was extracted. As shown in Figure 2A, RNA integrity decreased at ambient temperature over time for each formulation. The EDTA-based formulation, for which only a storage temperature of 4°C was tested, was superior to the formaldehyde-based formulations and yielded the best stability data even after 7 days at 4°C, as indicated by consistent RIN values above 8, which stayed almost unchanged from the RIN value obtained with immediate processing. The following experiments were conducted with EDTA-based formulation tubes.

The total RNA integrity of all other isolated cell types could be preserved, when stored at 4°C for up to 7 days in an EDTA-based additive (Figure 2B). The RIN value remained consistent for total RNA of CD19+ and CD14+ cells, but decreased slightly for CD8+ and CD15+ cells whilst remaining at a value, which is still acceptable for gene expression experiments. While the RNA integrity remained at a high level over a period of 7 days, the RNA yield decreased, as shown in Figure 2C. CD4+ and CD14+ cell preparations gave the highest total RNA yields, CD19+ and CD15+ the lowest.

### Experimental procedures do not activate cells

**Figure 3:**
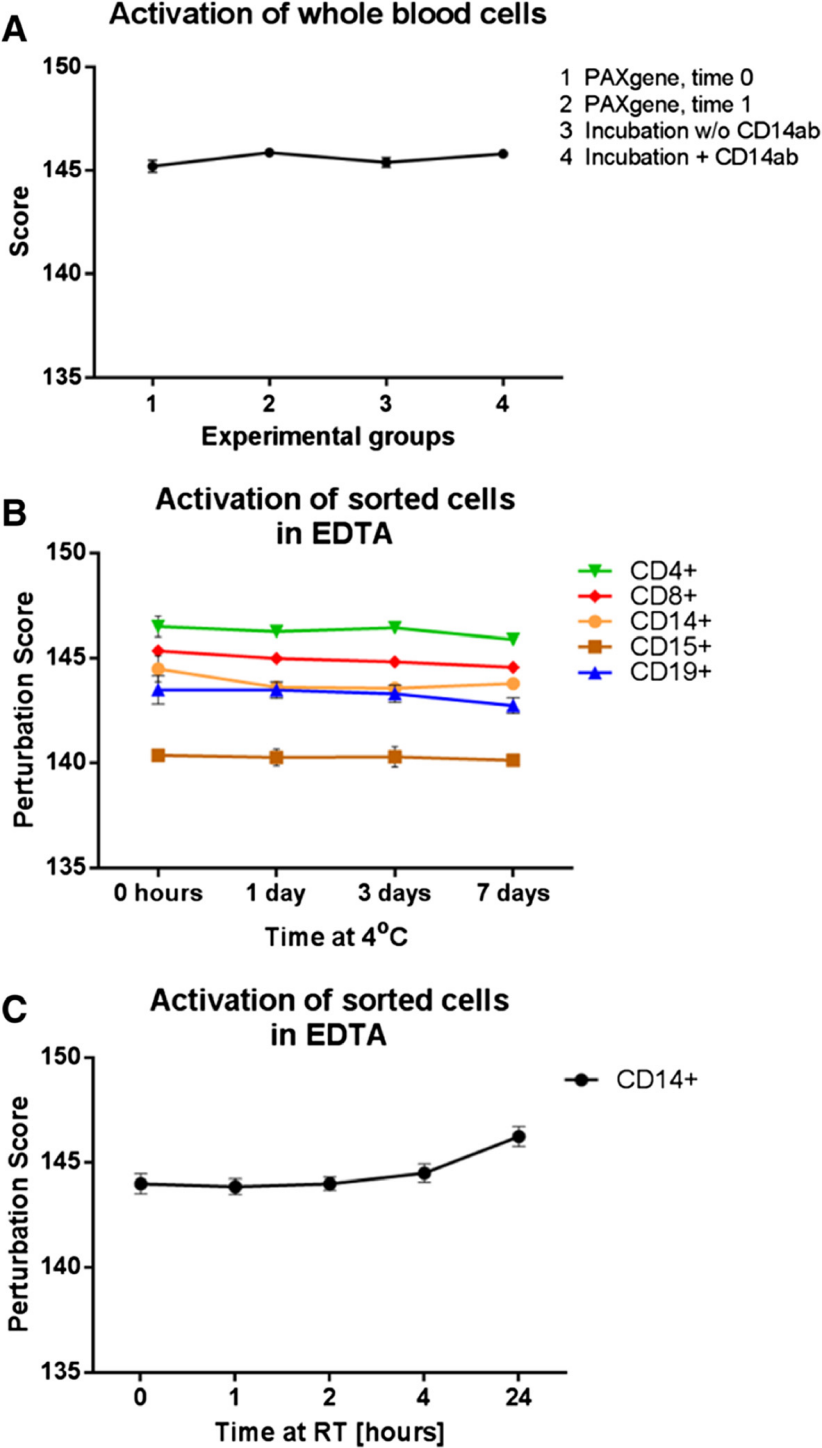
**Cells are not activated by experimental procedure. (A)** Four experimental groups (EG) were designed to test whether whole blood cells are activated at time of blood draw and transfer to PAXgene Blood RNA tubes (EG 1), and after storage of blood in EDTA tubes for 20 minutes prior to transfer to PAXgene Blood RNA tubes (EG2). Additionally, whole blood cells, which had been exposed to EDTA, were incubated with CD14ab-coated magnetic beads (EG3) or without magnetic beads (EG4). The perturbation score is nearly constant for all conditions, indicating a lack of activation. Mean values per group and the standard error of mean are shown. **(B)** Separated cells were stored in EDTA for up to 7 days prior to sorting. The perturbation score level is different for each cell type, but does not change significantly per cell type, indicating that cells are not activated during the experiment including the sorting process. Mean values per group and the standard error of mean are shown. **(C)** Perturbation score analysis for CD14+ cells which had been stored in EDTA as room temperature (RT). The score increases after 4 hours storage time. The experiment was discontinued after 24 hours.

Gene expression data from sorted cells may be informative of the health status of a patient. To avoid any distortion of the information on a patient¿, it is important to ensure that the experimental procedure of blood sampling at a health center, storage and/or transport, cell sorting, and RNA extraction does not affect the activation state of the immune cells in a sample. An experiment was designed to address this question. The results are shown in Figure 3A. An aliquot of 2.5 mL whole blood was collected directly into PAXgene Blood RNA Tubes containing RNA-stabilizing solution (¿PAXgene, Time 0¿, experimental group 1). To simulate a sample transport from the bedside to the laboratory, another 2.5 mL aliquot of whole blood was collected into EDTA-tubes, stored for 20 minutes without RNA stabilization and then transferred to a PAXgene Blood RNA Tube (¿PAXgene, Time 1¿, experimental group 2). To test the influence of the cell sorting procedure on the activation state of the cells (exemplified on CD14+ cells), 2.5 mL aliquots of whole blood stored in EDTA for 20 minutes were exposed to magnetic beads coated with anti-CD14 antibody or incubated without magnetic beads (¿PAXgene 3¿, ¿PAXgene 4¿, experimental group 3 and 4, respectively). Following the incubation, the whole blood aliquots were transferred to PAXgene Blood RNA Tubes. Total RNA was extracted from all samples. This experimental design simulates the steps from blood draw (experimental group 1) to the laboratories and the effect of storage in EDTA tubes (experimental group 2), the effect of experimental procedures (experimental group 3) and the effect of antibody-coated beads (experimental group 4). To test the activation state of the cells, we selected transcript groups representative of cellular stress response, apoptosis, cell cycle, and hypoxia including genes of the MAPK pathway, Nfkb pathway, and TNF pathway (Additional file 2: Table S1). We invented and applied a so-called perturbation score, calculated as $\sqrt{{?}_{i=1}^{n}I^{2}}$ per sample, where *I* is the signal intensity per transcript (over all n?=?503 transcripts in the combined groups defined above). The perturbation score for whole blood RNA was unchanged in all four conditions, indicating that the cells were not activated during the experiment (Figure 3A). Sorted cells were not activated when stored in EDTA tubes for up to 7 days (Figure 3B). In contrast, an increase of the perturbation score was notable when CD14+ cells were stored at room temperature (RT) for longer than 4 hours, indicating activation of transcription of stress response genes (Figure 3C). In addition, the expression of 419 of 503 cell stress genes (83%) was significantly affected after 4 hours storage at RT (p-value?<?0.05, absolute fold change?>?1.5, data not shown). In contrast, storage of CD14+ cells in EDTA at 4°C did not lead to gene expression changes during the observation period of 7 days (Additional file 1: Figure S2). None of the 503 selected cell stress genes showed statistically significant changes in expression levels between 0 days and 7 days of storage (data not shown).

### Enrichment of cell types by MACS cell separation

Blood samples and corresponding sorted cells from a subset of donors (10 females, 13 males) were stored at 4°C until analysis within 6 hours after venipuncture on a MACSQuant Flow Cytometer (Miltenyi Biotec GmbH). The purity of the sorted cells was within the specifications of the vendor (Additional file 1: Table S3).

**Figure 4:**
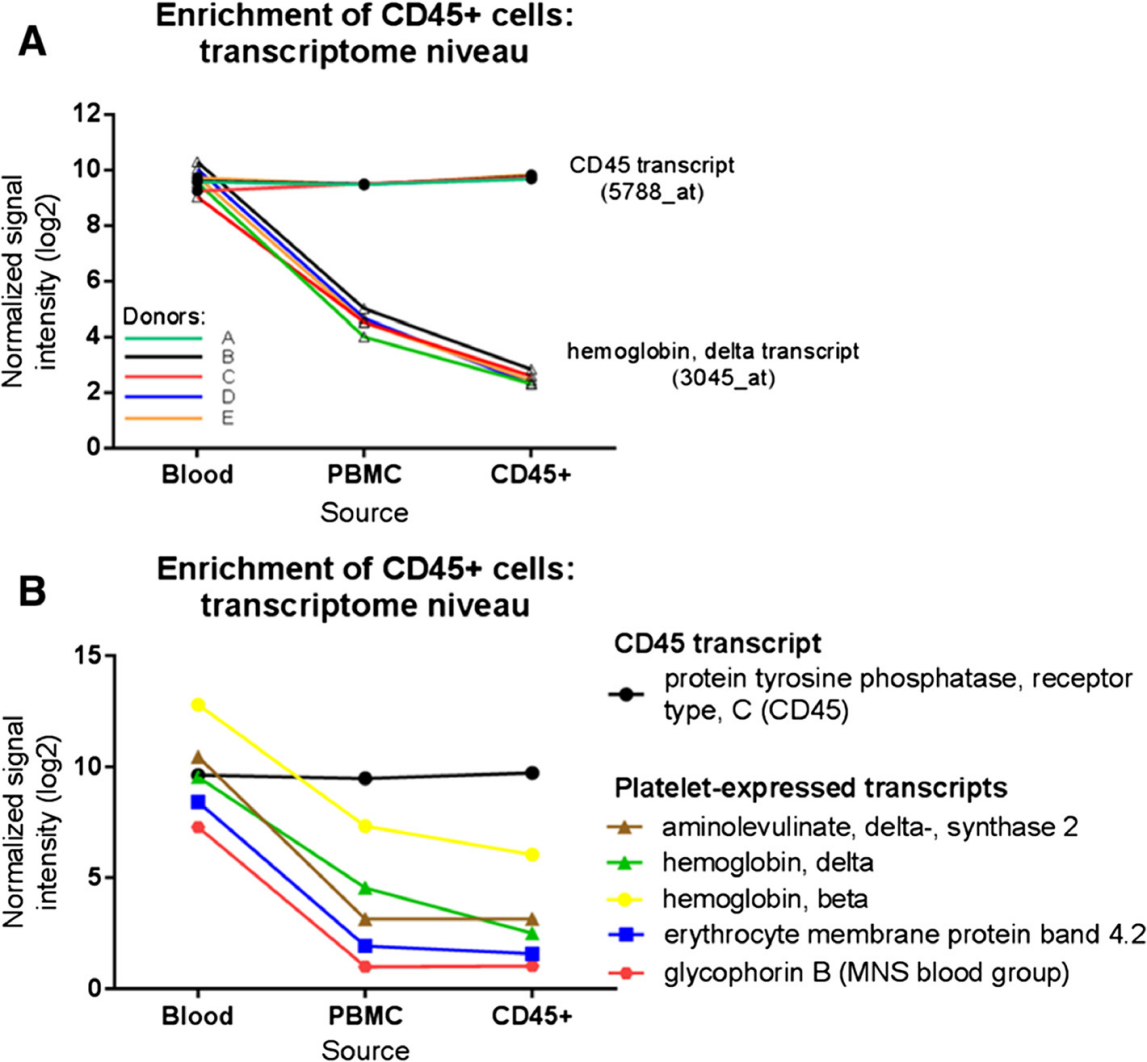
**Visualization of the enrichment of CD45+ cells.(A)** Expression profiles of CD45 and Hemoglobin delta from five donors. Expression levels indicate a 99% positive selection rate of CD45+ cells compared to blood, and a 97% enrichment compared to PBMC fractions. **(B)** The expression levels of platelet-expressed transcripts are markedly reduced in CD45+ samples, indicating an enrichment of CD45+ cells. Expression level reduction of genes expressed in blood but not in purified CD45+ cells indicate a 97% enrichment of CD45+. Mean values per group and the standard error of mean are shown.

Gene expression analysis was applied to estimate the enrichment of cells after cell sorting. As exemplified for CD45+ cells in Figure 4, the normalized signal intensities of the cell marker gene, in this case protein tyrosine phosphatase, receptor type C (PTPRC, CD45) did not change. In contrast, the expression levels for other genes, typically expressed in platelets, were strongly decreased in CD45+ cell preparations compared to whole blood. The reproducibility of the procedure we applied is illustrated in Figure 4A. The signal intensities of both the CD45 probe and the probe for hemoglobin delta (HBD) were consistent within independent preparations from five individual donors. To estimate the enrichment of CD45+ cells after MACS Cell Separation, we analyzed the significance of differences in signal intensities between CD45+ samples and whole blood samples by ANOVA. From the genes with the lowest p-value and highest absolute fold change, exemplary genes with platelet-specific expression were selected: hemoglobin delta (HBD), hemoglobin beta (HBB), aminolevulinate, delta-, synthase 2 (ALAS2), erythrocyte membrane protein 4.2 (EPB42), and glycophorin B (GYPB). All these genes had higher expression values in whole blood than in CD45+ cells (Figure 4B). To calculate the enrichment of CD45+ cells after MACS Cell Sorting, the mean signal intensity of the five platelet specific genes was calculated for each sample. The ratio of group mean values and the mean signal intensity of the CD45 probe (5788_at) indicated an enrichment of CD45+ cells of 99%. The data imply good coherence of cell population purity data calculated on the transcript level with those measured by flow cytometry, despite the application of different protocols.

### Gene expression analysis of isolated cell types

**Figure 5:**
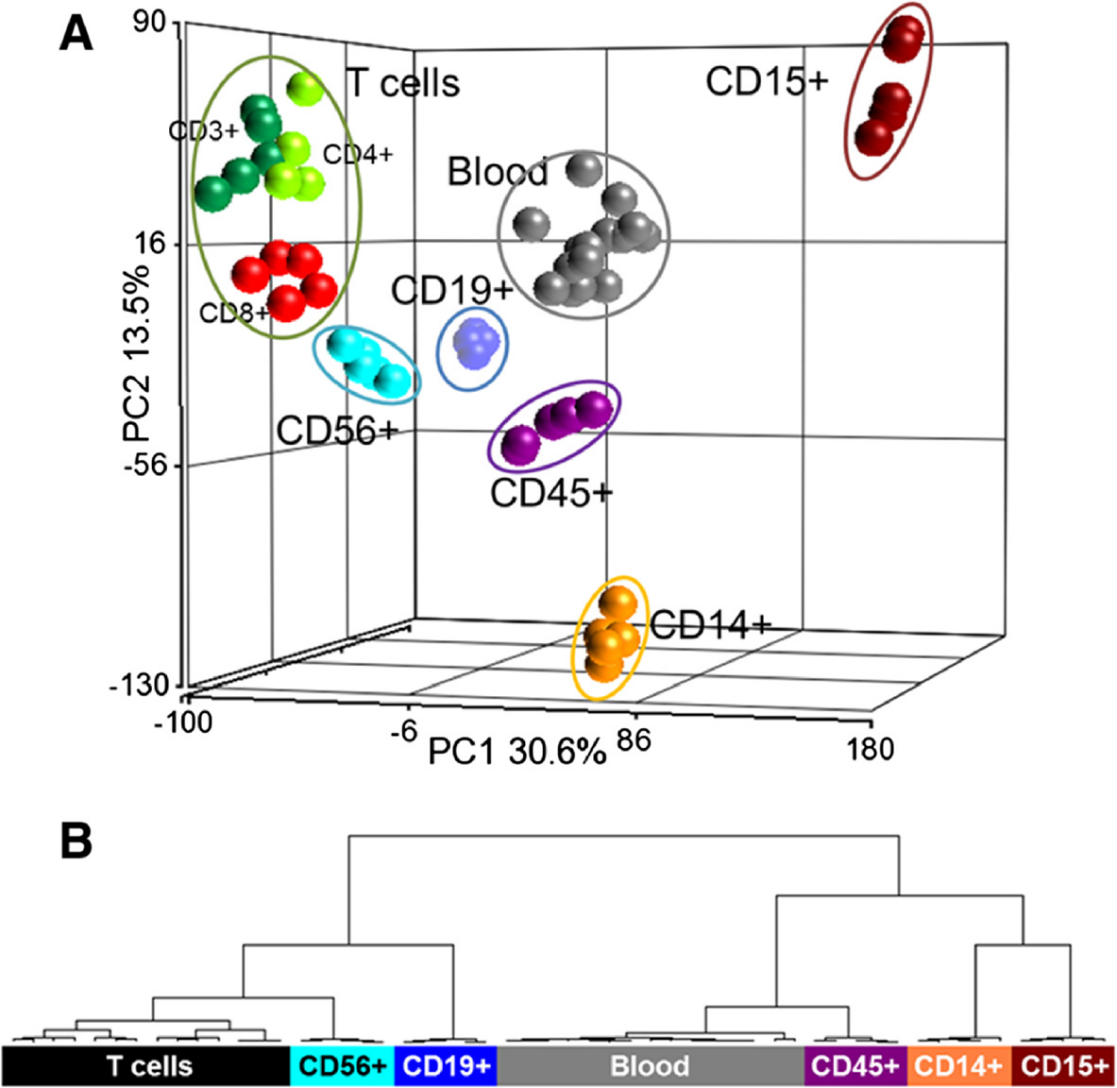
**Reproducibility and integrity of cell type preparations. (A)** Processed gene expression data of 8 separated cell types and of whole blood was visualized in a dimension reduction method, principal component analysis (PCA). Signal intensities from all 18898 probes separate cell types lead to a separation of cell types. T-cells (CD3+, CD4+ and CD8+) form one cluster which separates well from the B-cells (CD14+, CD15+, CD19+, CD45+, CD56+) or full blood. Each sphere represents a single sample. The x- and the y-axis (principal components 1 and 2, respectively) are labeled. The z-axis is not labled for graphical reasons. **(B)** Unsupervised hierarchical cluster analysis, same samples and gene list as in **(A)**. Cluster was generated with Euclidean distance and Ward¿s method. Only sample dendrogram is shown. CD3+, CD4+, and CD8+ are grouped as ¿T cells¿.

Gene expression data from isolated cell types can be used to address questions of basic biological relevance and for pharmacological research or biomarker development. Samples from eight isolated cell types and whole blood (total number of samples N?=?54) were subjected to gene expression analyses. Principal component analysis (PCA) is a computational data dimension reduction method and was applied to visualize the relative similarities of samples of different cell types and replicate groups. As shown in Figure 5A, differences in gene expression lead to a segregation of cell types and a clustering of samples by cell type when the data from all genes were used. As expected, T cell populations, i.e. CD3+, CD4+, and CD8+ cells formed a T cell cluster but maintained their individual subtype identity within this cluster. CD56+ natural killer cells, are located nearest to the CD3+, CD4+, CD8+ T cell cluster. Other cell types are separate from the T cell cluster and whole blood cells. Similarly, hierarchical cluster analysis with all genes reveals the close relationship of CD56+ cells to the T cell group, consisting of CD3+, CD4+, and CD8+ cells (Figure 5B). As in the PCA, the closeness of the replicates for a particular cell type is indicative of the good reproducibility of the experiment.

**Figure 6:**
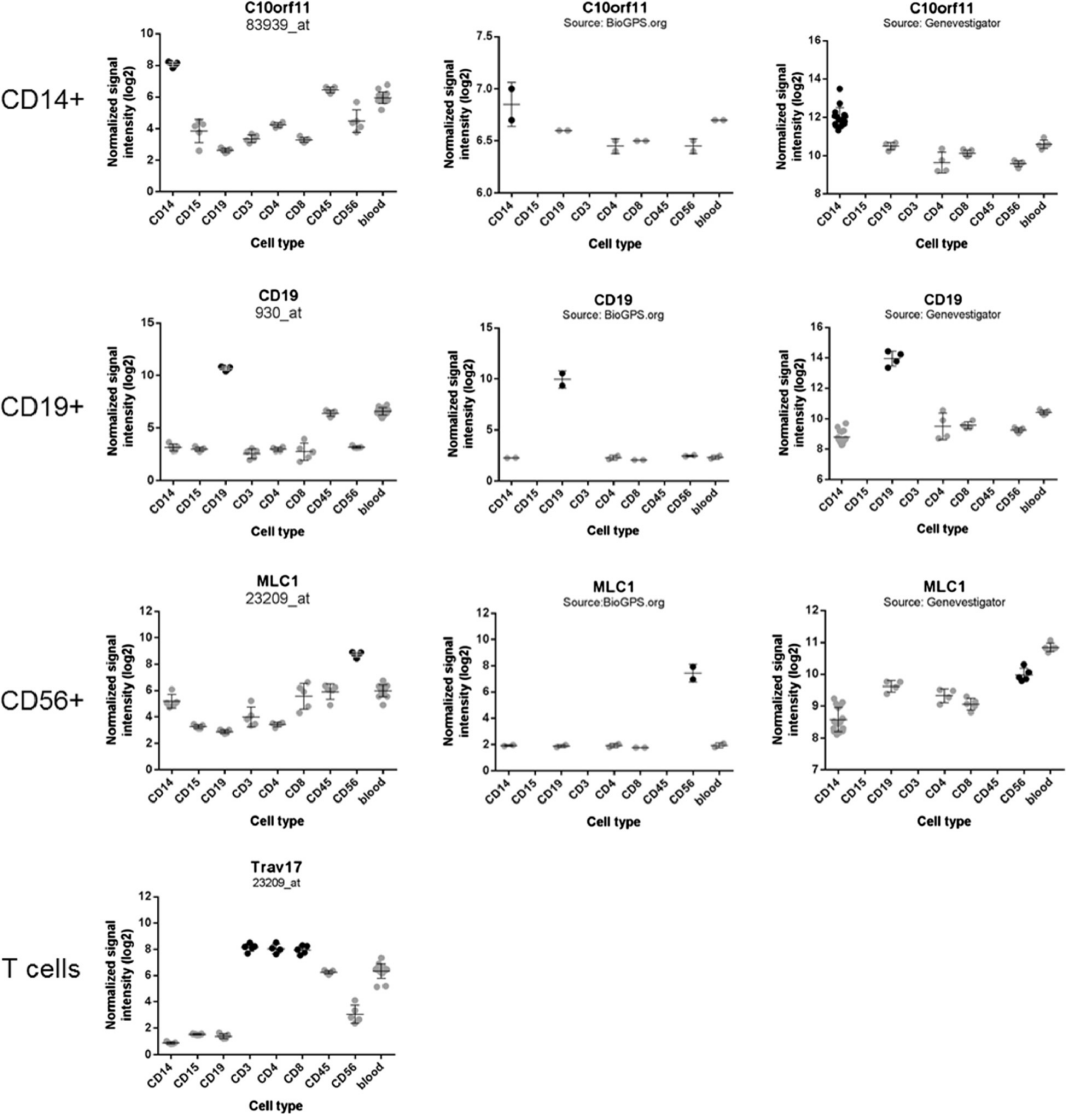
**Cell type specific gene expression.** Identification of cell type specific gene expression was performed by applying stringent filter on the gene expression data (for details see Materials and Methods). Some of the results are shown. The databases BioGPS (www.BioGPS.org) and the tool Genevestigator (www.genevestigator.com) were drawn upon to compare results of the present study with third party data. The present dataset shows that cell separation provides known and novel information on gene expression. Expression patterns of C10orf11 (CD14+ cells), CD19 (CD19+ cells), and MLC1 (CD56+ cells) was largely confirmed. No information was available for CD15+ cells in the external databases, and there was no information on TRAV17, which was highly expressed in T cell subtypes.

To test whether the present dataset can be used for the identification of genes with restricted expression signatures, signal intensity data was subjected to stringent filtering schemes. Expression in one cell type and not in the others was defined as a signal intensity ?200 in at least all but one replicates of one cell type, and ?100 in at least all but one of the other samples (except whole blood). The cutoff of 100 was set as upper background range level. CD19+ cells had the largest number of specific genes (n?=?98), followed by CD15+ (n?=?59) and CD14+ (n?=?33). No genes were identified whose expression was restricted to CD3+, CD4+ or CD8+ cells. When all three groups were combined in the filter approach, high expression levels were found for T cell receptor alpha variable 17 (TRAV17) in the three T cell subtypes. Some of the data are depicted in Figure 6. The gene expression database BioGPS (http://www.BioGPS.org/) and the software Genevestigator (http://www.Genevestigator.com) served as platforms to obtain third-party gene expression information. Besides other functionalities, the BioGPS project provides a reference gene expression atlas for a variety of species, organs and isolated cell types [33]. As shown in Figure 6, the trend towards specific expression of C10orf11 in CD14+ cells was confirmed in BiogGPS and Genevestigator. The gene CD19 was expressed exclusively in CD19+ B lymphocytes. This was confirmed in the other databases. Also high expression of MLC1 (megalencephalic leukoencephalopathy with subcortical cysts 1), a gene with so far unknown gene function, was detected in CD56+ cells. There were no data available for T cell receptor alpha variable 17 (TRAV17), a gene with expression limited to CD3+, CD4+, and CD8+ T cells in our dataset. No data were available for CD15+, CD3+, or blood-born CD45+ cells in the public databases.

## Discussion

Despite methodological improvements in the development of qualified, clinically relevant genomics biomarkers with non- or minimally invasive technologies [34]-[36], the path from discovery and validation, to regulatory approval, acceptance, and clinical application remains a challenge [37]-[39]. The analysis of genomics data of blood samples is demanding, due to various potentially confounding factors which need to be taken into account during the analytical procedure, including i) relative blood cell-type composition, ii) variation in cell-type gene expression signatures, iii) abundance of globin mRNA which interferes with microarray signal intensities, and iv) physiological condition of the individual [16],[40],[41], to name only some. The present project aimed to evaluate whether gene expression analysis of cells separated directly from whole blood by magnetic cell sorting (MACS Technology) was feasible. After evaluating the effects of several experimental conditions on RNA quality, and yield, followed by gene expression analysis of eight separated human blood cell populations, we concluded that MACSCell Separation can be combined with gene expression profiling and is well suited for processing of clinical trial samples. We found that EDTA-based anticoagulants perform much better than formaldehyde-based additives in terms of total RNA yield, integrity, and stability. The EDTA blood collection tubes for molecular assays gave the best results in terms of total RNA quality and quantity. In our hands, one of the formaldehyde-based tube-type, designed for stabilizing white blood cells for FACS analysis, gave suboptimal total RNA quality scores independent of the storage temperature and therefore we no longer evaluated those collection tubes for our purpose.

It is known that nucleic acids from formalin-fixed, paraffin-embedded (FFPE) tissues are usually significantly degraded and chemically modified by formaldehyde caused by the fixation and embedding conditions [42]. Therefore, nucleic acids isolated from FFPE samples are often of a lower molecular weight than those obtained from fresh or frozen samples. The other evaluated collection tube, designed for stabilizing circulating tumor cells, gave good quality total RNA when stored at 4°C for 3 days but the total RNA yields decreased and the transcripts were not preserved as well as in the PAXgene Blood RNA Tubes. These collection tubes with formaldehyde-based additives were therefore not suitable for the purpose of stabilizing the total RNA in this context.

RNA quality and yield were stable at a high level when stored at 4°C throughout the observation period of up to 7 days. Neither EDTA nor the addition of magnetic beads activated the cells. Lyons et al. investigated the effect of positive and negative selection by MACS Cell Separation on the activation state of cells [23]. They reported that positive selection did not activate the leukocyte subsets, supporting the notion presented here, that cells were not activated by storage or by MACS Cell Separation. We determined the status of activation by a series of experiments and the invention and application of a so called perturbation score, which compresses normalized signal intensities of pre-defined, study-relevant gene-groups into a single value. An unchanging perturbation score indicated lack of activation. The concept of gene scores to reflect severity of gene expression changes is not new. Adopted by others, Famulski et al. have successfully applied a gene score to clinical transplantation research and use it as molecular diagnostic tool [43]-[45]. Famulski¿s gene score is based on fold change and is thus dependent on reference gene expression data, for example from control samples. In contrast, the perturbation score we applied in the present study is independent of comparator data. Thus, individual samples can be assessed for expression changes of predefined gene groups.

Computational methods offer alternatives to physical cell separation. However, changes in cell composition and microenvironmental changes may represent obstacles towards the investigation of gene expression changes within a sample [46]. Several algorithms aiming to deconvolute cell composition have been developed [47]-[49]. Some algorithms rely on the *a priori* knowledge of either the cell type composition in a sample or on pre-determined gene expression profiles from isolated cell types [47],[49]. Recently, the possibility to reconstruct cell type composition in complex tissues without prior knowledge of either cell frequency or of pre-established cell type specific gene expression signatures was investigated on simulated data, awaiting further validation [50]. Other efforts aim to deconvolute cell composition changes caused by micro-environmental changes such as cell infiltration during progression of some diseases, or change of gene expression profiles during disease treatment. Further advances in this area, including validation, using data derived from clinical samples, will be highly welcome. Physical cell separation has limitations of its own. MACS Cell Separation can only be performed on cells in suspension, and only by employing predefined cell surface markers or marker combinations, excluding the discovery of new cell populations in a sample. However, due to the high technical reproducibility and robustness, MACS Cell Separation coupled to transcriptomics presents a valuable platform for routine investigations in clinical trials.

## Conclusions

Here we outline a workflow for magnetic bead-based, delayed cell sorting of CD3+ T-lymphocytes, CD4+ helper T-cells, CD8+ cytotoxic T-cells, CD14+ monocytes, CD15+ granulocytes, CD19+ B-lymphocytes, CD45+ leukocytes, and CD56+ NK cells, designed for transcriptome analysis of multi-center clinical trial samples. The observed cell-stability at 4°C provides the potential to uncouple the blood draw at the clinical site from MACS Cell Separation, e.g. at a central laboratory. Cells were not activated by the MACS process. This process facilitates the integration of cell sorting directly from whole blood coupled to transcriptomics in clinical trial protocols.

## Abbreviations

CDF: Chip description file

EDTA: Ethylenediaminetetraacetic acid

FACS: Fluorescence-activated cell sorting

HCHO: Formaldehyde

RIN: RNA integrity number

RNA: Ribonucleic acid

## Competing interests

The authors declare that they have no competing interests

## Authors¿ contributions

ML, AS wrote the manuscript, SSS, ML carried out the cell sorting and extraction experiments. NH carried out the gene expression experiments. MB carried out the hematology. FM carried out the FACS analysis and participated in the study design. AV carried out the FACS analysis. EL, MS and AS performed the statistical analysis. BG, UJ, EL, FS, ML participated in the study design. AV, NN and KJJ supported the project. All authors read and approved the final manuscript.

## Additional files

## Supplementary Material

Additional file 1: Supplementary material. Click here for file

Additional file 2: Supplementary table. Click here for file
