# Supplementary material for: Gene expression profiling of immunomagnetically separated cells directly from stabilized whole blood for multicenter clinical trials
Source: Clin Transl Med. 2014 Nov 13;3:36. doi: 10.1186/s40169-014-0036-z (PMC4424390; doi:10.1186/s40169-014-0036-z)
Supplement: Additional file 1: — Supplementary material. [file s40169-014-0036-z-S1.docx]

**Supplementary material:**

**Gene expression profiling of immunomagnetically separated cells directly from stabilized whole blood for multicenter clinical trials**

**Letzkus M et al**

**Methods**

**Magnetic bead-based cell sorting**

MACS® Cell Separation Technology is based on small superparamagnetic microbeads that are bound to a highly specific antibody against a particular cell marker, thus allowing for magnetic labeling of individual cell types. Separation occurs in a MACS Column which induces a high-gradient magnetic fields (~ 0.6 Tesla) when placed in a MACS® Separator.

The desired cells, collected in K_2_-EDTA anticoagulated whole blood samples, were magnetically labeled with Whole Blood MicroBeads (ø 50 nm) according to the manufacturer’s recommendations (Miltenyi Biotec GmbH, Bergisch Gladbach, Germany). The cell suspension was then applied to an autoMACS Pro Separator (Miltenyi Biotec GmbH) for fully automated separation, yielding the unlabeled negative fraction, which was discarded, and the magnetically labeled positive fraction.. No additional whole blood sample preparation such as density gradient centrifugation or erythrocyte lysis was required.

The collected positive cell fraction was pelleted at 300xg for 5 minutes. One aliquot of the final cell pellet from selected samples was used for FACS analysis and the remaining cell pellet was lysed directly in 350 µL RLT buffer containing 1% beta-mercaptoethanol from the RNA extraction kit (Qiagen, Hilden, Germany).

**Total RNA extraction**

The total RNA from the sorted and lysed cells was isolated with the RNeasy Mini Kit (Qiagen, Hilden, Germany) according to the manufacturer’s recommendations. After ethanol was added to the sorted cells to adjust binding conditions, the lysate was applied to an RNeasy spin column where RNA was selectively bound to the silica-gel membrane, contaminants including small RNA passed through. After an on-column DNase digestion, the remaining contaminants were removed in three efficient wash steps and the RNA was then eluted.

The total RNA from whole blood was isolated with the PAXgene Blood RNA Kit (Qiagen, Hilden, Germany) according to the manufacturer’s procedure. First, the nucleic acids in the PAXgene Blood RNA Tube were precipitated, washed and incubated in optimized buffers together with Proteinase K to digest proteins. An additional centrifugation (4400 g for 10 minutes) removed residual cell debris. After ethanol was added to adjust binding conditions, the lysate was applied to a PAXgene RNA spin column where RNA was selectively bound to the silica-gel membrane as contaminants passed through. After an on-column DNase digestion, the remaining contaminants were removed in three efficient wash steps and the RNA was then eluted.

The total RNA was quantified by its absorbance at λ = 260 nm (A260nm) on a Nanodrop spectrophotometer (Thermo Fisher Scientific Inc., MA, USA) and the purity was estimated from its A260nm/A280nm ratio. RNA integrity was assessed on an Agilent Bioanalyzer 2100 system using the Agilent RNA Pico Chip Kit (Agilent Technologies, Inc., Santa Clara, California, USA) or on a Caliper LabChip GX system (PerkinElmer, Waltham, Massachusetts, USA). RNA quality is reported as RNA integrity number (RIN, ([Schroeder, Mueller et al. 2006](#_ENREF_1))).

**Supplementary Table 1: provided in separate file**

**Supplementary Table 2: Hematology**

Hematology of blood samples of a subset of subjects (10 females, 13 males). WBC = White blood cells (x 1E3 cells / μL); RBC = Red blood cells (x 1E6 cells / μL); HGB = Hemoglobin (g / dL); HCT = Hematocrit (%); PLT = Platelets (x 1E3 cells / μL); Neut = Neutrophil cells (x 1E3 cells / μL or %); Lymph = Lymphocytes (x 1E3 cells / μL or %); Mono = Monocytes (x 1E3 cells / μL or %); Eos = Eosinophil cells (x 1E3 cells / μL or %); Baso = Basophil cells (x 1E3 cells / μL or %). N, number of samples; mean, mean count of cells x1E3 per µL blood volume; S.D., standard deviation; CV, coefficient of variation; median, median count of cells x1E3 per µL blood volume.

**Supplementary Table 3: Enrichment of cell types following MACS cell sorting**

| **Cell type** | **Mean purity (%)** | **S.D.** | **CV%** | **N** |
| --- | --- | --- | --- | --- |
| CD3 | 97.6 | 2.4 | 2.5 | 5 |
| CD4 | 94.6 | 7.1 | 7.5 | 5 |
| CD8 | 95.7 | 4.2 | 4.3 | 5 |
| CD15 | 98.8 | 1.1 | 1.1 | 5 |
| CD19 | 86.1 | 8.5 | 9.8 | 5 |
| CD45 | 91.6 | 5.1 | 5.6 | 5 |

Cell purities after cell sorting assessed by flow cytometry (exemplary). Mean purity (%), number of cells with desired characteristic vs all cells in sample preparation; S.D., standard deviation; CV, coefficient of variation; N, number of samples

**Supplementary Figure 1: Cell sorting experiments - RNA yield and quality**


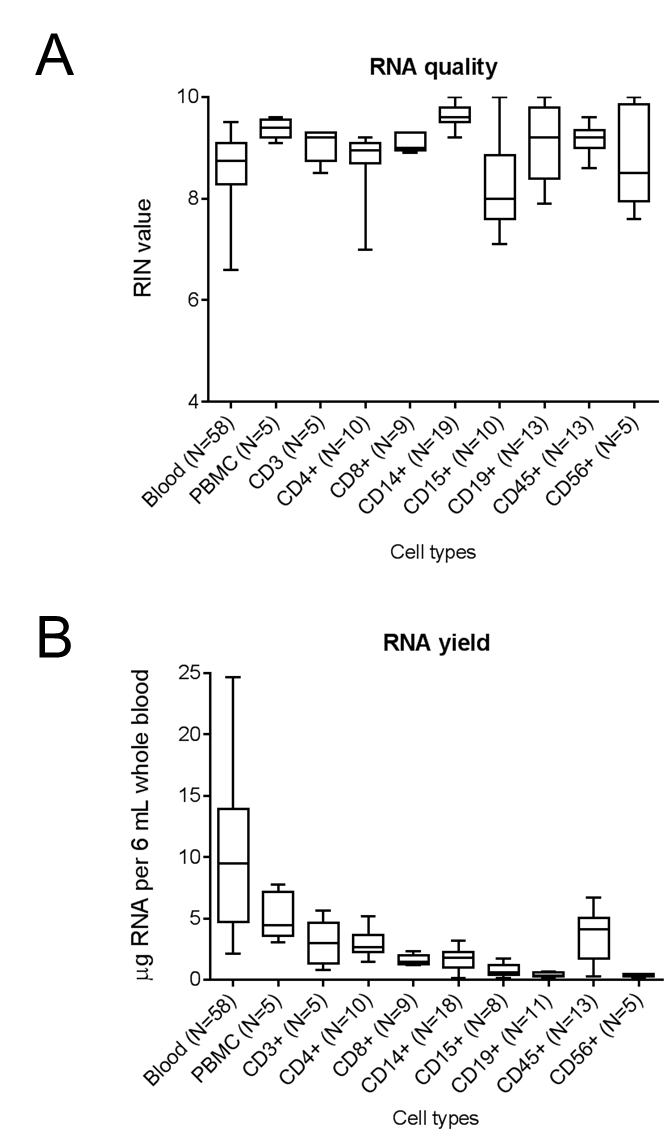


Supplementary Figure 1: RNA quality and yield

1. The RNA quality of the whole blood and sorted cell samples. RIN = RNA Integrity Number.
2. Total RNA yields from 6 mL whole blood. The total RNA was quantified by its absorbance at λ = 260 nm. Yields were corrected for the different blood collection volumes.

Samples from various stages of the project were considered for this analysis and do not in all cases correspond to the number of samples used in the expression analysis. Whiskers show the minimum to maximum range. The top and the bottom of the box are the 75^th^ and the 25th percentiles, respectively. The line inside the box represents the median.

**Supplementary Figure 2: Experimental procedures do not activate cells**


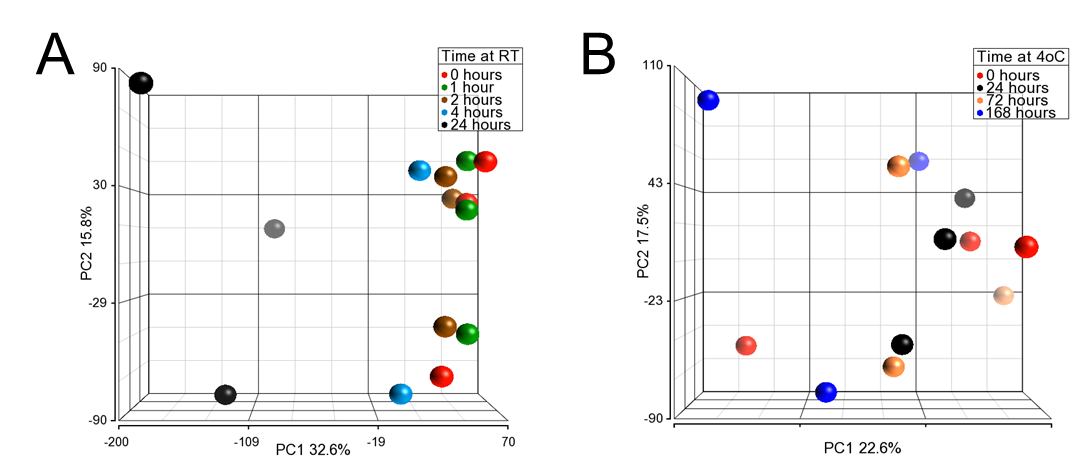


Supplementary Figure 2: Visualization of the influence of storage temperature on cell activation signatures.

1. Principal component analysis (PCA) of CD14+ samples, stored in EDTA at room temperature for up to 24 hours. Based on 503 selected cell activation genes, a separation of sample groups by time point is notable.
2. PCA of CD14+ samples, stored in EDTA at 4^o^C for up to 168 hours. No separation by time point group is notable.

Each sphere represents a single sample. The x- and the y-axis (principal components 1 and 2, respectively) are labeled. The z-axis is not labelled for graphical reasons.

**Reference for supplementary material**

Schroeder, A., O. Mueller, et al. (2006). "The RIN: an RNA integrity number for assigning integrity values to RNA measurements." BMC Mol Biol **7**: 3.
